# The complete mitochondrial genome of the *Copadichromis mloto*

**DOI:** 10.1080/23802359.2016.1222253

**Published:** 2016-11-22

**Authors:** Da-Shi Qi, Jie Jiang, Mei Wang, Lian-Qin Zhang, Xiao-Jing Huang

**Affiliations:** Department of Genetics, Xuzhou Medical University, Xuzhou, Jiangsu, People’s Republic of China

**Keywords:** *Copadichromis mloto*, genome, phylogenic relationship

## Abstract

The present study reported the complete mitochondrial genome of *Copadichromis mloto* for the first time. The mitochondrial genome of *C. mloto* possesses 16,583bp in length, involving 22 transfer RNA genes, 2 ribosomal RNA, 13 protein-coding genes and a control region. In addition, its GC content is 45.94% that is similar to that of *C. virginalis* (the GC content of 45.74%). Based on the complete mitochondrial genomes of 13 closely related species, the phylogenetic tree was further made to show their phylogenic relationship. The results will serve as a useful dataset for studying the evolution of Cichlidae mitochondrial genome.

The *Copadichromis mloto* is a member of Cichlidae native to the Southeast Arm of Lake Malawi (14°06'46.9”S 35°07'05.6”E). Our present study reported the complete mitochondrial genome of *C. mloto* for the first time, which would facilitate our understanding of the genetics, systematics and phylogenetic relationships of the many species of Cichlidae family.

The complete mitochondrial genome of *C. mloto* (Genbank accession KX196155) was assembled based on its raw sequences of whole genome (Genbank accession ERP002088). The whole genome of this species was sequenced by the Wellcome Trust Sanger Institute (SC) in the project PRJEB1254 (NCBI accession number), and the accession number of the sample on NCBI is SAMEA1877497. All the reads were mapped to a full mitochondrial genome reference sequences of *C.* virginalis (Genbank accession: NC_029761) by using SOAPaligner/soap2 (V2.21). Then we assembled the reads which could map to the reference genome by SPAdes3 (V3.1.0), and get the circular mitochondrial genome. Additionally, its complete mitochondrial genome sequence was also annotated by using DOGMA (Alverson et al. 2010).

The entire mitochondrial genome of *C. mloto* possesses 16,583 bp, involving 22 transfer RNA (tRNA) genes, 2 ribosomal RNA (rRNA) genes, 13 protein-coding genes (PCGs) and a control region. 28 genes including 12 PCGs, 14 tRNA and 2 rRNA are H-strand, whereas 9 genes including 1 PCG and 8 tRNA are L-strand. 11 PCGs had ATG start codon except for cox1 with GTG and nd6 with TTA. All PCGs in *C. mloto* stopped with TAG or TAA except for cox2 and nd4 with AGA and nd6 with CAT. The 22 tRNA genes begin from 67 bp (tRNA^Ser^, tRNA^Cys^) to 74bp (tRNA^Leu^), while 16S rRNA possesses 1676 bp and 12S rRNA possesses 941 bp in length. In addition, its GC content is 45.94% (27.49% A, 26.58% T, 30.12% C, and 15.82% G) that is similar to that of *C. virginalis* (the GC content of 45.74%). Based on the complete mitochondrial genomes of the *C. mloto* and other 12 closely related species, we also used MEGA6.06 to construct the phylogenetic tree by Maximum likelihood method (Figure 1) (Stamatakis et al. 2002; Tamura et al. 2013; Qi et al. 2015). Those results would facilitate our understanding of the evolution of Cichlidae mitochondrial genome.

**Figure 1.**
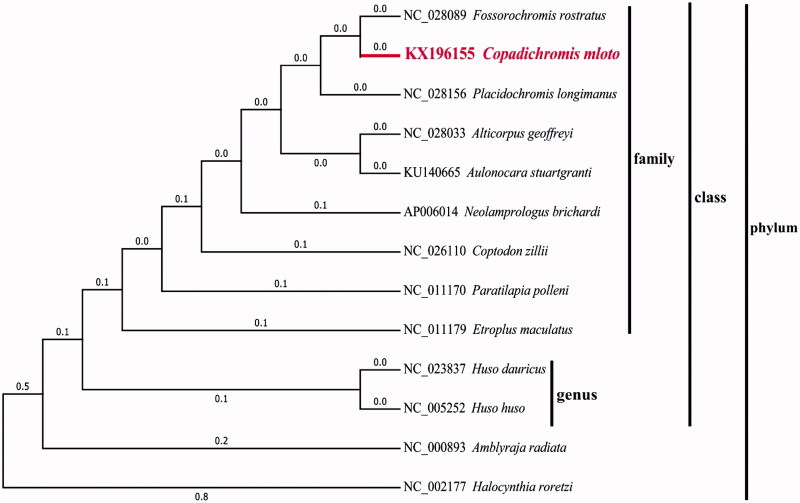
Maximum likelihood tree of complete mitochondrial genome of *C. mloto* and 12 other closely species, which have complete mitochondrial genome sequences in NCBI. The numbers in front of the species are genbank accession numbers.
